# *Fomitiporia rhamnoides* sp. nov. (Hymenochaetales, Basidiomycota), a new polypore growing on *Hippophae* from China

**DOI:** 10.3897/mycokeys.36.25986

**Published:** 2018-07-13

**Authors:** Tie-Zhi Liu, Qian Chen, Mei-Ling Han, Fang Wu

**Affiliations:** 1 College of Life Sciences, Chifeng University, Chifeng, Inner Mongolia 024000, China; 2 Institute of Microbiology, Beijing Forestry University, Beijing 100083, China; 3 College of Life Science, Langfang Normal University, Langfang 065000, China

**Keywords:** Hymenochaetaceae, taxonomy, wood-inhabiting fungi

## Abstract

Based on morphology and phylogenetic analysis, *Fomitiporia rhamnoides* sp. nov. is described. It is characterised by perennial, pileate basidiomata, distinctly shining poroid surface, a zonate context, 11–13 pores per mm, parallel tramal hyphae and regularly arranged contextual hyphae, the presence of cystidioles, globose, hyaline, thick-walled, smooth, dextrinoid, strongly cyanophilous basidiospores measuring 5.8–7 × 5.4–6.5 µm and growing on *Hippophae rhamnoides* in northern China. *Fomitiporia rhamnoides* differs from other *Fomitiporia* species growing on *Hippophae* by its smaller pores (11–13 per mm vs. <10 per mm).

## Introduction

*Fomitiporia* Murrill (Murrill 1907), typified by *F. langloisii* Murrill, is an important genus in Hymenochaetaceae because some species are pathogens of trees (Dai et al. 2007, Rajchenberg and Robledo 2013, Ota et al. 2014) whereas some other species are claimed to be medicinal (Dai et al. 2009). *Fomitiporia* is easy to distinguish from other members of Hymenochaetaceae in having subglobose to globose, hyaline, thick-walled, strongly dextrinoid and cyanophilous basidiospores (Fiasson and Niemelä 1984, Amalfi and Decock 2013, Chen and Cui 2017).

During investigations on wood-inhabiting fungi in northern China, in Hebei and Shanxi provinces, some specimens of a *Fomitiporia* species were collected on living *Hippophae rhamnoides*. They are characterised by distinctly small pores which make them different from other *Fomitiporia* species growing on *Hippophae*.

To understand their taxonomic placement, phylogenetic analysis was carried out based on the nuc rDNA regions of the 5.8S rDNA (ITS) and nuc 28S rDNA D1-D2 domains. Molecular analyses showed that the sampled specimens are clustered into a lineage representing an unknown species of *Fomitiporia*.

## Materials and methods

The studied specimens are deposited at the herbarium of the Institute of Microbiology, Beijing Forestry University (BJFC). The microscopic procedure follows Zhou et al. (2016a). The following abbreviations are used: IKI = Melzer’s reagent, IKI− = both inamyloid and indextrinoid, KOH = 5% potassium hydroxide, CB = Cotton Blue, CB+ = cyanophilous, CB− = acyanophilous, L = mean spore length (arithmetic average of all spores), W = mean spore width (arithmetic average of all spores), Q = variation in the L/W ratios between the specimens studied and n = number of spores measured from a given number of specimens. Special colour codes followed Petersen (1996).

CTAB rapid plant genome extraction kit-DN14 (Aidlab Biotechnologies Co. Ltd, Beijing) was used to obtain PCR products from dried specimens according to the manufacturer’s instructions with some modifications. Two DNA gene fragments, ITS and 28S were amplified using respectively the primer pairs ITS5/ITS4 (White et al. 1990) and LR0R/LR7 (http://www.biology.duke.edu/fungi/mycolab/primers.htm). The PCR procedures for ITS and 28S followed Zhou et al. (2016b). DNA sequencing was performed at the Beijing Genomics Institute and newly generated sequences were deposited in the GenBank database.

Sequences generated for this study and additional sequences downloaded from GenBank were aligned using BioEdit (Hall 1999) and ClustalX (Thompson et al. 1997).

In the study, nuclear ribosomal RNA genes were used to determine the phylogenetic position of the new species. *Phellinus uncisetus* Robledo, Urcelay & Rajchenb. was designated as an outgroup following Decock et al. (2007).

Maximum parsimony analysis was applied to the combined dataset of ITS+28S sequences using PAUP* version 4.0b10 (Swofford 2002). All characters were equally weighted and gaps were treated as missing data. Trees were inferred using the heuristic search option with TBR branch swapping and 1000 random sequence additions. Max-trees were set to 5000, branches of zero length were collapsed and all parsimonious trees were saved. Clade robustness was assessed using bootstrap analysis with 1000 replicates (Felsenstein 1985). Descriptive tree statistics tree length (TL), consistency index (CI), retention index (RI), rescaled consistency index (RC) and homoplasy index (HI) were calculated for each maximum parsimonious tree generated. The Maximum likelihood (ML) tree was constructed using raxmlGUI 1.2 (Stamatakis 2006, Silvestro and Michalak 2012) with GTR+I+ G model and auto FC option (Pattengale 2010) in bootstrap (BS) replicates.

MrModeltest 2.3 (Posada and Crandall 1998, Nylander 2004) was used to determine the best-fit evolution model for the combined dataset of ITS+28S sequences for running Bayesian inference (BI). BI was calculated with MrBayes 3.1.2 (Ronquist and Huelsenbeck 2003). Four Markov chains were run for two runs from random starting trees for 2 million generations for the combined dataset of ITS+28S sequences and trees were sampled every 100 generations. The first quarter of the generations were discarded as burn-in. The majority rule consensus tree of all remaining trees was calculated. Branches that received bootstrap support for Maximum parsimony (BP), Maximum likelihood (BS) and Bayesian posterior probabilities (BPP) greater than or equal to 50% (BP/BS) and 0.95 (BPP), respectively, were considered as significantly supported.

## Phylogeny results

**Figure 1.**
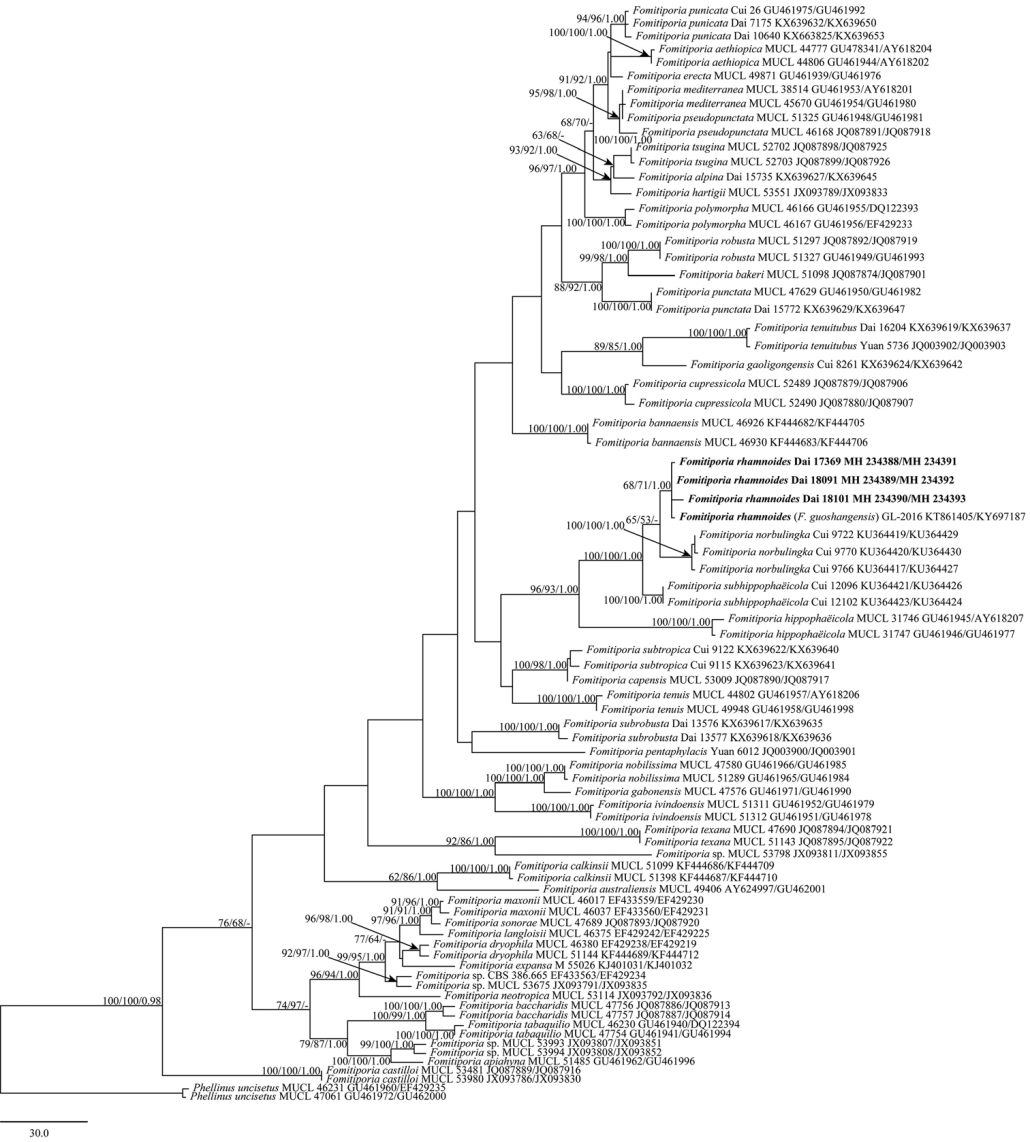
Phylogenetic tree inferred from maximum parsimony (MP) analysis based on the combined dataset of ITS and 28S. Only maximum parsimony (BP), maximum likelihood (BS) and Bayesian posterior probabilities (BPP) greater than or equal to 50% (BP), 50% (BS) and 0.95 (BPP) are reported on the branches.

The combined ITS+28S dataset includes 78 specimens and resulted in an alignment of 1737 characters, of which 1124 characters are constant, 98 are variable and parsimony-uninformative and 515 are parsimony-informative. Maximum parsimony analysis yielded 28 equally parsimonious trees (TL = 1515, CI = 0.549, HI = 0.451, RI = 0.813, RC = 0.446). The best model for the combined dataset, estimated and applied in the Bayesian analysis, is GTR+I+G, lset nst = 6, rates = invgamma; prset statefreqpr = dirichlet (1,1,1,1). Bayesian analysis and ML analysis resulted in a similar topology to MP analysis, with an average standard deviation of split frequencies = 0.007191 (BI). Therefore, only the MP tree was presented and BP, BS and BPP values simultaneously above 50%, 50% and 0.95, respectively, were indicated at the nodes (Fig. 1). The phylogeny shows that the three newly sequenced specimens gathered with *F. guoshangensis* S. Guo & L. Zhou in a single, isolated, variably supported (68%/71%/1.00) clade (Fig. 1).

### Taxonomy

#### Fomitiporia rhamnoides

Taxon classificationFungiHymenochaetalesHymenochaetaceae

T.Z. Liu & F. Wu sp. nov.

825105

Figs 2 , 3

##### Holotype.

CHINA. Hebei Province, Zuolu County, Xiaowutai Nature Reserve, Shanjiankou, on living tree of *Hippophae rhamnoides*, 10.IX.2017, *Dai 18091* (BJFC025621).

##### Etymology.

*Rhamnoides* (Lat.) refers to the species growing on *Hippophae rhamnoides*.

Basidiomata perennial, pileate, solitary or a few imbricated, hard corky and without odour or taste when fresh, woody hard and medium in weight when dry; pilei dimidiate to ungulate, triquetrous in section, projecting up to 5 cm, 7 cm wide and 2.5 cm thick at base; pileal surface yellowish-brown, greyish-brown to dark brown, concentrically sulcate, at first velutinate, becoming glabrous and slightly cracked with age; margin obtuse. Poroid surface clay-buff to yellowish-brown when fresh, becoming orange brown to snuff brown when dry, shining; sterile margin yellowish-brown, up to 3 mm wide; pores circular, 11–13 per mm, dissepiments entire. Context yellowish-brown, zonate, woody hard, up to 1.5 cm thick; tubes greyish-brown, paler than context, hard corky to brittle, up to 1 cm long, annual layers indistinct.

**Figure 2.**
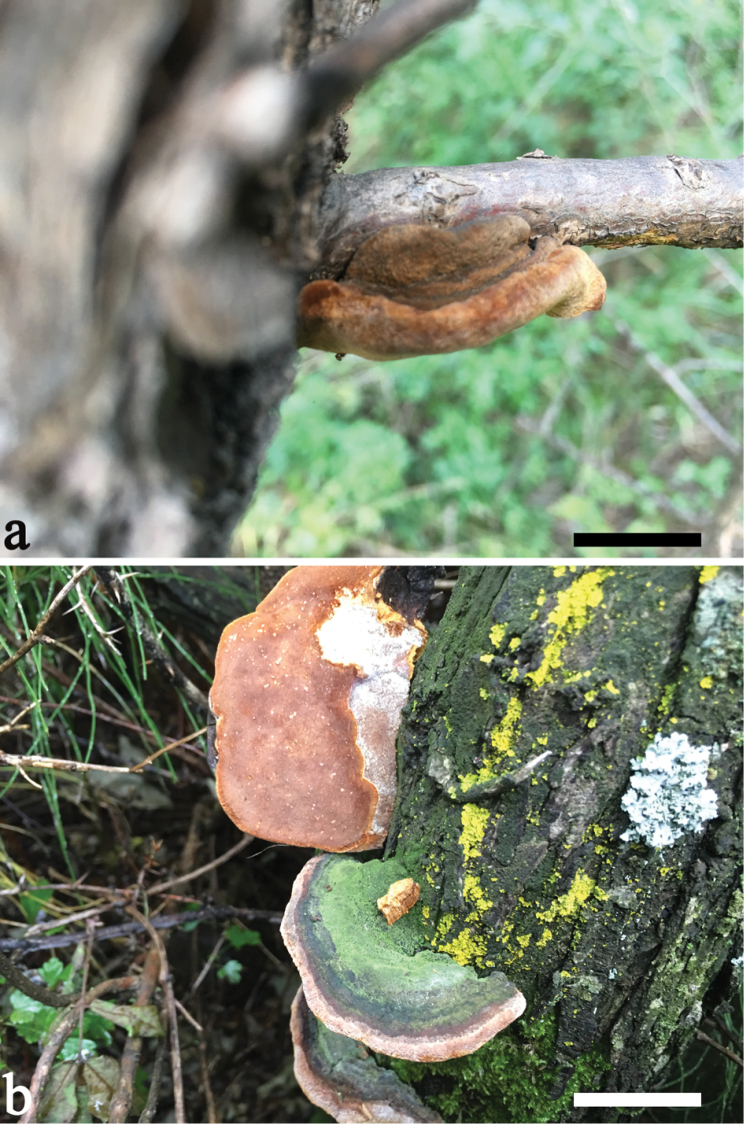
Basidiocarps of *Fomitiporia rhamnoides* (**a** Dai 18087 showing a juvenile basidiomata **b** Dai 18100 showing the mature basidiomata; Scale bars: 3 cm).

##### Hyphal structure.

Hyphal system dimitic; generative hyphae simple septate; tissue darkening but otherwise unchanged in KOH.

##### Context.

Generative hyphae hyaline to pale yellow, thin- to slightly thick-walled, occasionally branched, frequently septate, 3–4 µm in diam., skeletal hyphae yellowish-brown, thick-walled with a wide lumen, unbranched, occasionally septate, straight, regularly arranged, 4.5–6 µm in diam.

##### Trama of the tubes.

Generative hyphae hyaline to pale yellowish, thin-walled, occasionally branched, frequently septate, 2–3 µm in diam., skeletal hyphae dominant, yellowish-brown, thick-walled with a wide lumen, unbranched, occasionally septate, straight, parallel along the tubes, 2.5–4 µm in diam. Setae absent; cystidioles present, more or less ventricose, hyaline, thin-walled, 12–20 × 3–6 μm; basidia subglobose to barrel-shaped, with four sterigmata and a simple septum at the base, 8–16 × 6–10 µm; basidioles dominant in hymenium, in shape similar to basidia, but slightly smaller; big rhomboid crystals present amongst hymenium.

##### Spores.

Basidiospores globose, hyaline, thick-walled, smooth, dextrinoid in Melzer’s reagent, strongly CB+, (5.2–)5.8–7(–7.3) × (5–)5.5–6.5(–6.8) µm, L = 6.47 µm, W = 6.06 µm, Q = 1.06–1.08 (n=60/2).

##### Additional specimens (paratypes) examined.

CHINA. Hebei Province, Zuolu County, Xiaowutai Nature Reserve, Shanjiankou, on living tree of *Hippophae rhamnoides*, 10.IX.2017, *Dai 18087* (BJFC025617), *Dai 18088* (BJFC025618), *Dai 18090* (BJFC025620), *Dai 18100* (BJFC025630), *Dai 18101* (BJFC25631). Shanxi Province, Zuoyun County, Santun, on living tree of *Hippophae rhamnoides*, 19.V.2017, *Dai 17368* (BJFC024903), *Dai 17369* (BJFC024904), *Dai 17370* (BJFC024905).

##### Type of rot.

Causing a white rot.

**Figure 3.**
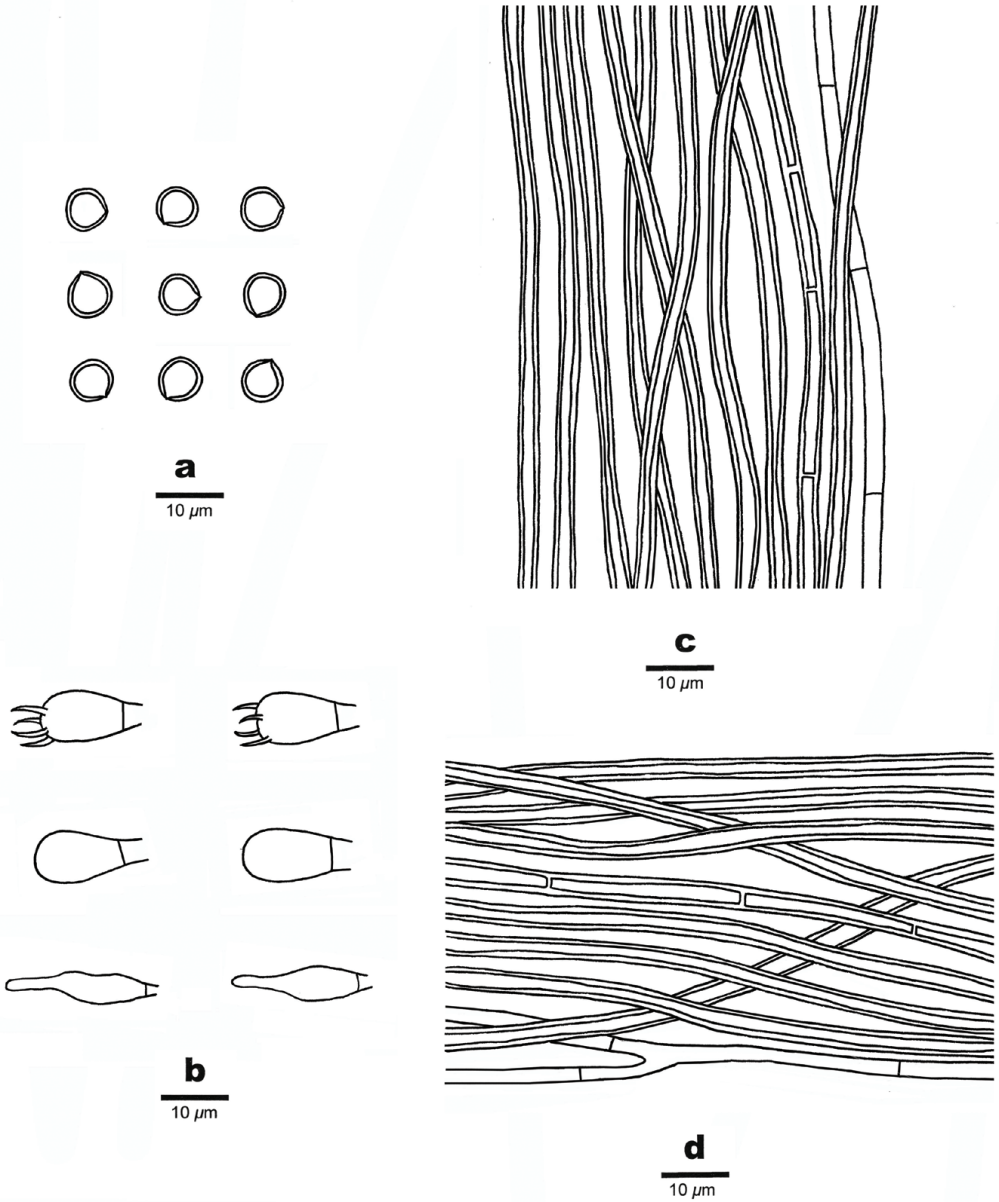
Microscopic structures of *Fomitiporia rhamnoides* (from the holotype). **a** Basidiospores **b** Basidia, basidioles and cystidioles **c** Hyphae from trama **d** Hyphae from context.

## Discussion

*Fomitiporia rhamnoides* is characterised by its very small pores (11–13 per mm) and growing on *Hippophae rhamnoides*. It has the same sequences of *Fomitiporia guoshangensis*, an illegitimate name (art. 7, 8, 32A, code of nomenclature) also described based in Chinese collections (Guo et al. 2016).

Three species of *Fomitiporia*, *F. hippophaëicola* (H. Jahn) Fiasson & Niemelä, *F. norbulingka* B.K. Cui & Hong Chen, *F. subhippophaëicola* B.K. Cui & H. Chen, have been reported on *Hippophae* (Chen et al. 2016, Chen and Cui 2017, Ryvarden and Melo 2017). Amongst them, *F. hippophaëicola* has a distribution in Europe whereas *F. norbulingka* and *F. subhippophaëicola* have, so far, been found in Tibet, China (Chen et al. 2016). *Fomitiporia hippophaëicola* was previously recorded in China (Dai 2010), but the voucher specimens were re-identified as *F. subhippophaëicola*. The main characters of *F. hippophaëicola*, *F. norbulingka* and *F. subhippophaëicola* were given by Chen et al. (2016).

*Fomitiporia rhamnoides* resembles *F. hippophaëicola*, *F. norbulingka* and *F. subhippophaëicola* by sharing similar basidiomata and basidiospores, but it can be distinguished from these three species by smaller pores (11–13 per mm, vs. 5–7 per mm in *F. hippophaëicola*, 6–9 per mm in *F. norbulingka*, 8–10 per mm in *F. subhippophaëicola*). Phylogenetically, *F. rhamnoides* forms a single lineage and is closely related to *F. norbulingka*.

*Fomes yasudae* Lloyd was originally described from Japan on an angiosperm trunk (Lloyd 1915) and Ryvarden (1989) considered it as a synonym of *Fomitiporia robusta* (P. Karst.) Fiasson & Niemelä. *Fomes yasudae* may be confused with *Fomitiporia rhamnoides* because of its small pores, but it has distinct smaller basidiospores (3.5–4 µm in diam.) and uncracked upper surface (Lloyd 1915). *Fomes yasudae* is most probably an independent species rather than *Fomitiporia robusta* because the latter has larger basidiospores (5.8–7.3 × 5.3–6.8 µm, Niemelä 2005).

## Supplementary Material

XML Treatment for Fomitiporia rhamnoides

## References

[B1] Amalfi M Decock C (2013) *Fomitiporia castilloi* sp. nov. and evidence for multiples clades around *F. apiahyna* in Meso- and South America, representing potential species. Mycologia 105: 873–887. 10.3852/11-423 23709522

[B2] Chen H Cui BK (2017) Multi-locus phylogeny and morphology reveal five new species of *Fomitiporia* (Hymenochaetaceae) from China. Mycological Progress 16: 687–701. 10.1007/s11557-017-1306-0

[B3] Chen H Zhou JL Cui BK (2016) Two new species of *Fomitiporia* (Hymenochaetales, Basidiomycota) from Tibet, southwest China. Mycologia 108: 1010–1017. 10.3852/16-011 27474517

[B4] Dai YC (2010) Hymenochaetaceae (Basidiomycota) in China. Fungal Diversity 45: 131−343. 10.1007/s13225-010-0066-9

[B5] Dai YC Cui BK Yuan HS Li BD (2007) Pathogenic wood-decaying fungi in China. Forest Pathology 37: 105–120. 10.1111/j.1439-0329.2007.00485.x

[B6] Dai YC Yang ZL Cui BK Yu CJ Zhou LW (2009) Species diversity and utilization of medicinal mushrooms and fungi in China (Review). International Journal of Medicinal Mushrooms 11: 287–302. 10.1615/IntJMedMushr.v11.i3.80

[B7] Decock C Herrera FS Robledo G Castillo G (2007) 10.3852/mycologia.99.5.733

[B8] Felsenstein J (1985) Confidence intervals on phylogenetics: an approach using bootstrap. Evolution 39: 783–791. 10.2307/2408678 28561359

[B9] Fiasson JL Niemelä T (1984) The Hymenochaetales: a revision of the European poroid taxa. Karstenia 24: 14–28. 10.29203/ka.1984.224

[B10] Guo S Pang R Guo LH Li YT Xu LN Nan XJ Liu XG (2016) Analysis on nutrition and acute toxicity of a new species of the *Fomitiporia* sp. Edible Fungi of China 35(4): 54–57.

[B11] Hall TA (1999) BioEdit: a user-friendly biological sequence alignment editor and analysis program for Windows 95/98/NT. Nucleic Acids Symposium Series No. 41: 95−98.

[B12] Lloyd CG (1915) Synopsis of the genus *Fomes*. Mycological Writings 4: 209–288.

[B13] Niemelä T (2005) Polypores, lignicolous fungi. Norrlinia 13: 1–320.

[B14] Nylander JAA (2004) MrModeltest v2. Program distributed by the author. Evolutionary Biology Centre, Uppsala University, Uppsala.

[B15] Ota Y Hattori T Nakamura H Terashima Y Lee SS Miyuki Y Sotome K (2014) Taxonomy and phylogenetic position of *Fomitiporia torreyae*, a causal agent of trunk rot on Sanbu-sugi, a cultivar of Japanese cedar (*Cryptomeria japonica*) in Japan. Mycologia 106: 66–76. 10.3852/13-045 24396106

[B16] Pattengale ND Alipour M Bininda-Emonds ORP Moret BME Stamatakis A (2010) How many bootstrap replicates are necessary? Journal of Computational Biology 17: 337–354. 10.1089/cmb.2009.0179 20377449

[B17] Petersen JH (1996) Farvekort. The Danish Mycological Society´s color-chart. Foreningen til Svampekundskabens Fremme, Greve.

[B18] Posada D Crandall KA (1998) Modeltest: Testing the model of DNA substitution. Bioinformatics 14: 817–818. 10.1093/bioinformatics/14.9.817 9918953

[B19] Rajchenberg M Robledo G (2013) Pathogenic polypores in Argentina. Forest Pathology 43: 171–184. 10.1111/efp.12032

[B20] Ronquist F Huelsenbeck JP (2003) MrBayes 3: Bayesian phylogenetic inference under mixed models. Bioinformatics 19: 1572–1574. 10.1093/bioinformatics/btg180 12912839

[B21] Ryvarden L (1989) Type studies in the Polyporaceae 21. Species described by C.G. Lloyd in *Cyclomyces*, *Daedalea*, *Favolus*, *Fomes* and *Hexagonia*. Mycotaxon 35: 229–236

[B22] Ryvarden L Melo I (2017) Poroid fungi of Europe, 2nd edition. Synopsis Fungorum 37: 1–431.

[B23] Silvestro D Michalak I (2012) raxmlGUI: a graphical front-end for RAxML. Organisms Diversity & Evolution 12: 335–337. 10.1007/s13127-011-0056-0

[B24] Stamatakis A (2006) RAxML-VI-HPC: maximum likelihood-based phylogenetic analyses with thousands of taxa and mixed models. Bioinformatics 22: 2688–2690. 10.1093/bioinformatics/btl446 16928733

[B25] Swofford DL (2002) PAUP*: Phylogenetic analysis using parsimony (*and other methods). Version 4.0b10. Sinauer Associates, Sunderland, Massachusetts.

[B26] Thompson JD Gibson TJ Plewniak F Jeanmougin F Higgins DG (1997) The Clustal_X windows interface: flexible strategies for multiple sequence alignment aided by quality analysis tools. Nucleic Acids Research 25: 4876−4882. 10.1093/nar/25.24.4876 PMC147148 9396791

[B27] White TJ Bruns TD Lee S Taylor J (1990) Amplification and direct sequencing of fungal ribosomal RNA genes for phylogenetics. In: Innis MA, Gelfand DH, Sninsky JJ, White TJ (Eds) PCR protocols, a guide to methods and applications. Academic, San Diego, 315−322. 10.1016/B978-0-12-372180-8.50042-1

[B28] Zhou LW Vlasák J Decock C Assefa A Stenlid J Abate D Wu SH Dai YC (2016a) Global diversity and taxonomy of the *Inonotus* linteus complex (Hymenochaetales, Basidiomycota): *Sanghuangporus* gen. nov., *Tropicoporus excentrodendri* and *T. guanacastensis* gen. et spp. nov., and 17 new combinations. Fungal Diversity 77: 335–347. 10.1007/s13225-015-0335-8

[B29] Zhou LW Vlasák J Qin WM Dai YC (2016b) Global diversity and phylogeny of the *Phellinus igniarius* complex (Hymenochaetales, Basidiomycota) with the description of five new species. Mycologia 108: 192–204. 10.3852/15-099 26553776

